# Non-invasive monitoring of microbial triterpenoid production using nonlinear microscopy techniques

**DOI:** 10.3389/fbioe.2023.1106566

**Published:** 2023-02-28

**Authors:** Mariam Dianat, Ute Münchberg, Lars M. Blank, Erik Freier, Birgitta E. Ebert

**Affiliations:** ^1^ Institute of Applied Microbiology (iAMB), Aachen Biology and Biotechnology (ABBt), RWTH Aachen University, Aachen, Germany; ^2^ University Development and Strategy, Ruhr University Bochum, Bochum, Germany; ^3^ Interdisciplinary Center for Machine Learning and Data Analytics (IZMD), University of Wuppertal, Wuppertal, Germany; ^4^ Australian Institute for Bioengineering and Nanotechnology, The University of Queensland, Brisbane, QLD, Australia

**Keywords:** CARS microscopy, second harmonic generation, lipids, natural compounds, baker’s yeast, metabolic engineering

## Abstract

**Introduction:** Bioproduction of plant-derived triterpenoids in recombinant microbes is receiving great attention to make these biologically active compounds industrially accessible as nutraceuticals, pharmaceutics, and cosmetic ingredients. So far, there is no direct method for detecting triterpenoids under physiological conditions on a cellular level, information yet highly relevant to rationalizing microbial engineering.

**Methods:** Here, we show in a proof-of-concept study, that triterpenoids can be detected and monitored in living yeast cells by combining coherent anti-Stokes Raman scattering (CARS) and second-harmonic-generation (SHG) microscopy techniques. We applied CARS and SHG microscopy measurements, and for comparison classical Nile Red staining, on immobilized and growing triterpenoid-producing, and non-producing reference *Saccharomyces cerevisiae* strains.

**Results and Discussion:** We found that the SHG signal in triterpenoid-producing strains is significantly higher than in a non-producing reference strain, correlating with lipophile content as determined by Nile red staining. In growing cultures, both CARS and SHG signals showed changes over time, enabling new insights into the dynamics of triterpenoid production and storage inside cells.

## Introduction

**FIGURE 1:**
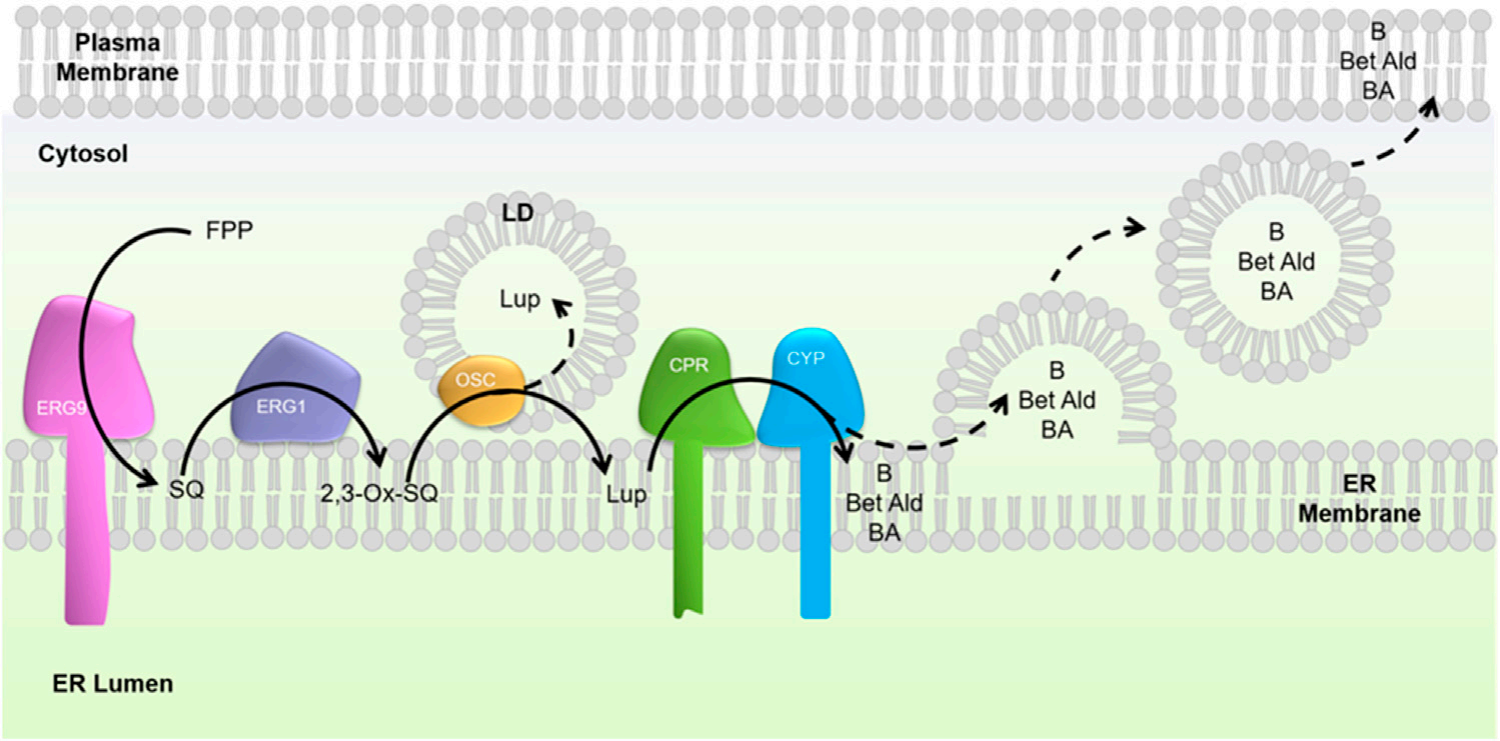
Schematic overview of the heterologous biosynthesis of lupane-type triterpenoids in *S. cerevisiae*. The native yeast metabolism converts farnesyl pyrophosphate (FPP) to squalene (SQ) by squalene synthase (Erg9). Squalene epoxidase (Erg1) catalyses the conversion of squalene to 2,3-oxidosqualene (OSQ). The heterologous pathway constitutes an oxidosqualene cyclase (OSC), catalyzing the cyclization of 2,3-oxidosqualene to lupeol, as well as a cytochrome P450 monooxygenase (CYP) and corresponding cytochrome P450 reductase (CPR), oxidizing lupeol to betulin (B), betulin aldehyde (Bet-Ald), and betulinic acid (BA). Dashed arrows correspond to a hypothetical transfer of triterpenoids from the endoplasmic reticulum (ER) membrane into lipid droplets (LDs) and further to the plasma membrane.

Triterpenoids are a large group of secondary plant metabolites with a wide application range in diverse consumer sectors (Tolstikov et al., 2005; Zhang et al., 2017; Gong et al., 2019). Extraction of triterpenoids from their natural source and their chemical synthesis is shown to be challenging and complicated due to their low concentration and complex structure (Jager et al., 2009), rendering the application of microbial cell factories for their production a promising alternative. The yeast *Saccharomyces cerevisiae* was shown to be a desirable host for producing triterpenoids due to its endogenous mevalonate and sterol pathways providing the common triterpenoid precursors squalene and 2,3-oxidosqualene (Figure 1), the presence of endoplasmic reticulum (ER) to anchor membrane-associated plant enzymes, and the plethora of genetic tools for metabolic engineering (Dai et al., 2013; Czarnotta et al., 2017). Because of their hydrophobicity, triterpenoids accumulate intracellularly, presumably in the ER membrane, where the last synthesis steps occur. However, considering the dynamic ER structure (English and Voeltz, 2013) and membrane trafficking between the ER, the cell membrane, lipid droplets, and other organelles, the post-synthesis localization of triterpenoids is unclear.

In addition to the unclear intracellular triterpenoid distribution between membranes and lipid droplets, there is little knowledge about triterpenoid production among individual cells of a population. Such insights would be valuable for understanding hydrophobic molecule trafficking and guiding strain engineering strategies.

Nile Red [9-diethylamino-5H-benzo (α) phenoxazine-5-one] is a lipophilic dye that is fluorescent in the presence of a hydrophobic environment, while the fluorescence is quenched in the presence of an aqueous medium (Greenspan et al., 1985). As a highly solvatochromic strain, the emission and excitation wavelengths of Nile red shift depending on the polarity of its environment (Plenderleith et al., 2014). This fluorescent dye is widely applied to detect lipid particles and lipid droplets. Furthermore, Nile red is applied for the detection of other hydrophobic compounds, such as microplastic (Tamminga, 2017), polyhydroxyalkanoates (PHAs) (Zuriani et al., 2013), and squalene (Choi et al., 2018). Due to the hydrophobic structure of triterpenoids, we assumed that Nile red staining is also a suitable method for detecting these compounds produced intracellularly by engineered yeast cells.

Coherent Anti-Stokes Raman Scattering (CARS) and Second Harmonic Generation (SHG) are nonlinear spectroscopy variants that deliver sub-micrometer resolution in a multimodal imaging setup. CARS is a nonlinear, non-destructive and nonperturbative (Evans and Xie, 2008; Okuno et al., 2010; Moura et al., 2016) variant of vibrational Raman spectroscopy, offering fast hyperspectral and chemically resolved imaging of biological samples (Potma and Xie, 2004; El-Mashtoly et al., 2014; Cheng and Xie, 2015; Ebersbach et al., 2018). CARS can be employed to investigate the distribution of cell components such as proteins or lipids (Rinia et al., 2008; Pliss et al., 2010; Meyer et al., 2012). Brackmann et al. applied CARS as a label-free and non-invasive method for imaging of lipid storage in *S. cerevisiae* (Brackmann et al., 2009). The applicability of CARS in identifying molecules in a complex sample depends on the analyte under investigation and the surrounding matrix (Meyer et al., 2012; Ebersbach et al., 2018). SHG is a nonlinear and non-destructive (Narice et al., 2016) optical process occurring in highly organized non-centrosymmetric objects and can be used for microscopic imaging (Sheppard et al., 1977; Pavone and Campagnola, 2013). It has been applied for imaging biological structures like membranes (Campagnola et al., 1999; Pavone and Campagnola, 2013) or protein aggregates (Roth and Freund, 1979; Chen et al., 2012) and might be applicable for imaging non-natural aggregations if these exhibit a highly organized non-centrosymmetric structure on the nanoscale. While CARS allows a rough identification of subcellular aggregates, SHG delivers information about the structural properties of these aggregates beyond the actual optical resolution (Clays et al., 2001; Chu et al., 2002). Further, these two non-invasive microscopy techniques do not alter the specimen by labeling or staining, making them highly valuable methods to study the physiological state of cells. The combination of both CARS and SHG is also of great potential, e.g. as shown for applications in biomedical imaging (Lee et al., 2015), detection of microbial systems in soil matrices (Lee et al., 2022), and visualization of carotenoids and starch granules in plant cells (Brackmann et al., 2011).

To our knowledge, no studies exist in which the combination of CARS and SHG is applied for the analysis of intracellular heterologous compounds synthesized in yeast. Therefore, we aimed to test the applicability of the combination of these non-invasive techniques to analyze triterpenoid and lipid accumulation in recombinant yeast cells on a qualitative level. As a model triterpenoid, we used the lupane-type triterpenoid betulinic acid (Hordyjewska et al., 2019) and its precursors, which are of huge interest for pharmaceutical applications due to potent anti-tumor and anti-(retro) viral activities (Seca and Pinto, 2018). We first applied Nile red staining to show that triterpenoid-producing strains differ from the reference strain regarding the accumulation of intracellular hydrophobic compounds. Next, we tested the applicability of CARS and SHG microscopy to monitor triterpenoid accumulation in recombinant yeast. We performed CARS and SHG analyses for both fixed and live (growing) cells to evaluate the applicability of both methods for determining intracellular triterpenoid accumulation with temporal and spatial resolution.

## Materials and methods

### Strains and media

*Saccharomyces cerevisiae* CEN. PK 113-17A (MATα, *ura3-52*; *leu2-3_112*; *TRP1*; *HIS3*; *MAL2-8C*; *SUC2*; EUROSCARF) and two strains, *S. cerevisiae* BA6 and *S. cerevisiae* BA6∆, originating from *S. cerevisiae* CEN. PK 102-5B (MATa*, ura3-52, his3-11, leu2-3/112, TRP1, MAL2-8c, SUC2*) and engineered for betulinic acid production were used in this study. Strain *S. cerevisiae* BA6 has been described in Guo et al. (2022); it expresses a truncated form of *HMG1* (Polakowski et al., 1998), a single copy of *Arabidopsis thaliana* ATR2 and multiple copies of *Vitis vinifera* CYP716A15 and *Olea europaea* OEW. All heterologous genes were integrated into chromosomes. In addition, lanosterol synthase Erg7p activity was downregulated by replacement of the native gene with an *ERG7* mutant, as described in Guo et al. (2022). *S. cerevisiae* BA6∆ (unpublished) is a *pah1* deletion mutant of BA6, generated through replacement of *PAH1* with a hygromycin resistance cassette (*YMR165C*∆:hphMX).

Cryogenic stocks were prepared by mixing equal volumes, 400 µL each, of a sterile glycerol (40% v/v) solution and a 40 h culture in mineral salt medium and stored at −80°C. 500 µL of a single cryogenic stock was used to inoculate a 50 ml pre-culture. All cultivations were performed in WM8+ (Czarnotta et al., 2017), a modified version of the mineral salt medium WM8 (Lang and Looman, 1995), complemented with vitamin and trace elements of Verduyn minimal medium (Verduyn et al., 1992), resulting in the following composition: (NH_4_)H_2_PO_4_ 0.25 g/L, NH_4_Cl 2.80 g/L, sodium glutamate 10 g/L, MgCl_2_·6H_2_O 0.25 g/L, CaCl_2_·2H_2_O 4.60 g/L, KH_2_PO_4_ 2 g/L, MgSO_4_·7H_2_O 0.55 g/L, myoinositol 100 mg/L, ZnSO_4_·7H_2_O 6.25 mg/L, FeSO_4_·7H_2_O 3.50 mg/L, CuSO_4_·5H_2_O 0.40 mg/L, MnCl_2_·4H_2_O 0.10 mg/L, MnCl_2_·2H_2_O 1.00 mg/L, Na_2_MoO_4_·2H_2_O 0.50 mg/L, nicotinic acid 11.00 mg/L, pyridoxin-HCl 26.00 mg/L, thiamin-HCl 11.00 mg/L, biotin 2.55 mg/L, calcium pantothenate 51.00 mg/L, Na_2_EDTA 2.92 g/L, CoCl_2_.6H_2_O 0.300 mg/L, H_3_BO_3_ 1.00 mg/L, KI 0.10 mg/L, and *p-*aminobenzoic acid 0.20 mg/L. For the reference strain, the WM8+ medium was complemented with 400 mg/L leucin and 400 mg/L uracil. Static cultivations for real-time microscopy measurements were performed with WM8+ medium complemented with 16 g/L ethanol as sole carbon source, which previous studies suggested as a preferred substrate for triterpenoid production (Czarnotta et al., 2017).

### Cultivation strategies

Batch cultivations in 500 ml shake flasks started with a filling volume of 20 ml. A pre-culture was used to inoculate the main cultures to a start optical density measured at 600 nm (OD_600_) of 0.1. The optical density of the cultures was determined using a spectrophotometer (Ultrospec 10, biochrom, Germany) with 0.5% phosphate-buffered saline (PBS) used as blank. A predetermined correlation factor of 0.29 g cell dry weight (CDW) per unit OD_600_ for strain BA6∆ and 0.2 for BA6 and the reference was used to derive the CDW concentration. The cultivations took place in a rotary shaker (New Brunswick Innova 44R) at 30°C, 150 rpm, and 25 mm shaking diameter. For microscopy measurements, samples were taken during exponential growth on glucose and after the diauxic shift during growth on ethanol.

**FIGURE 2:**
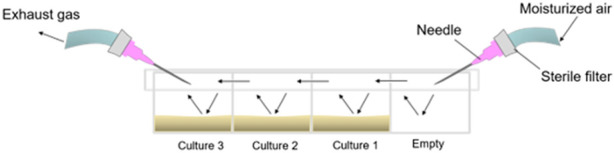
Experimental setup of static culture experiments. Static culture experiments took place in a glass-bottom microscopy chamber with a coverslip (ibidiplates μ‐Slide 4 Well Glass Bottom, ibidi GmbH Germany). Three of the four chambers were filled with 1 ml of the yeast culture at a starting OD600 of 0.4. The chamber was fitted onto the microscope directly below the objective lens to take microscopy images of each chamber at regular intervals. The cultures were aerated with ambient air to prevent oxygen limitation. To minimize evaporation, the incoming, sterile air was moisturized with water and ethanol by passing it through an ethanol:water solution (50:50 (v/v)). Two holes on both ends of the lid served as gas inlet and outlet; a third opening in the dividing wall ensured gas exchange between the two chambers. Arrows indicate airflow through the chambers.

For static, non-shaken cultivations, we designed the experimental setup shown in Figure 2.

### Quantification of triterpenoids

Samples of 800 μL of culture broth were transferred into 2 ml sample tubes for the analysis of intracellular triterpenoid concentrations and stored at −20°C until further processing or immediately analyzed. Cells were disrupted by adding 250 μL of glass beads (smooth 500 μm, Carl Roth, Karlsruhe, Germany), 80 μl 1 M HCl, and 800 μl chloroform:methanol (ratio 80:20, v/v) and vigorously agitating in the Mini-Beadbeater 16 (BioSpec Products, Bartlesville, United States) for 4 min. Samples were centrifuged for 10 min at 13,000 rpm (16,089 x g) and 5°C. 200 μl of the lower organic phase were transferred into a glass vial (VVial, clear, 9 mm, screw, Phenomenex) containing an insert (15 mm tip, wide opening, 0.2 mL, 6 × 31 mm, clear, Macherey-Nagel GmbH & Co. KG Germany) for High-Performance Liquid Chromatography (HPLC) analysis. Product quantification was conducted with a reverse HPLC system (Thermo Fisher UltiMate 3,000 Dionex). Samples were kept a 4°C in an autosampler (Thermo Fisher UltiMate 3,000). Five µL of the sample were injected into a C18 column (Merck Spherisorb ODS-2 (5 μm), 250 × 4 mm, Merck KGaA, Germany) with a guard column (EC 4/3 NUCLEODUR C18 Gravity, 3 μm, Macherey-Nagel GmbH & Co. KG Germany), while the column temperature was kept at 40°C in a thermostatic column compartment (Thermo Fisher UltiMate 3,000). Analytes were eluted using gradient chromatography at 1.2 mL/min flow rate using a binary pump (Ultimate 3,000 Pump, Dionex). Solvent A was acetonitrile (LC-MS grade). Solvent B was 0.2% (v/v) formic acid of high purity (≥98%). The linear gradient method was as follows: 20% B from 0 to 1 min, 20%–0% B from 1 to 13 min, 0% B held until 23 min, then back to 20% B from 23 to 26 min. Solvents were degassed using an online degasser. Analytes of interest were monitored using a charged aerosol detector (CAD, esa ERC GmbH) driven by a nitrogen generation system (Parker, BALSTON, Analytical Gas Systems). Technical duplicates were measured, i. e., two times 800 μl were taken from the same cultivation, processed, and analyzed. For quantification of triterpenoids, serial dilutions (df = 2) of standards for lupeol (Sigma Aldrich, ≥94%), betulin (Sigma Aldrich, ≥98%), betulinic acid (Sigma Aldrich, ≥98%), and betulin aldehyde (Cfm Oskar Tropitzsch GmbH, ≥98%) in chloroform were measured.

### Preparation of immobilized samples for microscopy

Yeast cells were immobilized on agarose beds for microscopic analyses. One ml of warm 1% agarose gel was applied onto a microscope slide and covered with a second microscopy slide to force the formation of a planar surface. After solidification, the upper slide was carefully removed. Ten µL of a culture diluted to an OD_600_ of 1.0 was applied on the agarose layer and protected with a cover glass. Desiccation of samples was prevented by covering the edges of the coverslip with clear lacquer and analyzing samples immediately.

### Nile Red staining

Nile Red staining was performed based on a protocol described in Zuriani et al. (2013). First, 1 mL of a cell culture, which had an OD_600_ adjusted to 1, was centrifuged at 12,000 g for 5 min. The supernatant was discarded, and the pellet was resuspended in 1 mL 0.5% PBS. Next, 60 μl of Nile red (80 μg/mL dissolved in acetone) was added to the cell suspension. The samples were incubated for 30 min at RT in the dark. After incubation, samples were centrifuged again at 12,000 g for 5 min, and the supernatant was discarded. Pellets were carefully resuspended in 1 mL 0.5% PBS, and 100 μl of the sample were transferred to a 96-well microplate. Fluorescence was measured at different excitation (Ex) and emission (Em) wavelengths using the Multi-Mode Microplate Reader Synergy; Ex/Em 550/638 nm for phospholipids, 515/583 nm for triglycerides, as recommended by the manufacturer. Data were analyzed with the software Gen5. All reads took place at RT (ca. 23°C) with the following parameters: read height 8 mm, read speed normal, delay 100 msec, measurements/data point 10, gain 100.

### Microscopy system setup

CARS and SHG measurements were performed with a modified Leica TCS SP8 CARS (Leica Microsystems CMS GmbH, Wetzlar, Germany) equipped with a picoEMERALD™ laser system (APE GmbH, Berlin, Germany). The CARS images were generated *via* a pump/probe beam at 816 nm and 817 nm and a Stokes beam at 1,064 nm while the SHG images were based on the pump/probe beam. CARS spectra were generated by tuning the pump/probe beam in a spectral window from 807 nm to 819 nm (3,000–2,800 cm^-1^) or 787 nm–826 nm (3,300–2,700 cm^-1^). The real-time growth experiments were performed using a Leica HC PL Fluotar 10x/0.30 DRY objective. All other measurements were performed using a Leica Fluotar VISIR 25x/0.95 WATER objective. The generated signals were detected by non-descanned Leica photomultipliers in EPI and forward direction. A more detailed description of the systems is given in Ebersbach et al. (2018).

### CARS and SHG analysis of pure standards

Pure lupeol, betulin, betulin aldehyde, betulinic acid, and ergosterol (Sigma Aldrich, ≥95%) were analyzed in crystalline conditions and squalene in liquid form. Saturated solutions of ergosterol and the main triterpenoid products, betulin and betulinic acid in ethanol were prepared to analyze the CARS and SHG spectra in their dissolved state. Assuming additivity of the signal intensities, the mean intensities of dissolved substances were corrected by subtracting the mean CARS signal intensity of pure ethanol. The difference in mean intensities is the estimated CARS signal caused by these substances. While betulin and betulinic acid generated only a weak CARS signal, ergosterol produced a stronger CARS signal in solution.

### Colocalization analysis

All images were re-scaled to equal contrast levels for proper quantification of the colocalization of CARS and SHG. The lower threshold was set equal to the value of the background signal of the respective channel. The upper threshold was chosen so that no oversaturation occurred. For CARS images, the lower contrast value was set to 20 and the upper value to 60; for SHG images, the lower contrast value to 7, and the upper to 30; and for transmission images, the lower value was 25 and the upper 100. An image gradient was calculated for re-scaled transmission images, and a threshold was set that defined which gradient values were considered for evaluation. From this gradient image, objects smaller than 100 pixels were removed since they are too small for cells and, most likely, debris. Subsequently, all holes in determined structures were filled. From the generated masks, the number of cells per object was determined manually and by comparison with the original transmission image. For each object within an image, the colocalization evaluation was performed separately, i.e., for each object the number of all pixels above a colocalization intensity threshold of 25% of the maximum intensity was determined for both channels (CARS and SHG). From these, the colocalizing pixels were determined within the object. The ratio of colocalization of CARS and SHG was calculated with Eqs 1, 2.

$$Ratio\;of\;SHG\;colocalizing\;with\;CARS=\frac{No.\;of\;pixels\;colocalizing\;in\;SHG\;and\;CARS\;channels}{Total\;number\;of\;pixels\;in\;CARS\;channel}$$ (1)

$$Ratio\;of\;CARS\;colocalizing\;with\;SHG=\frac{No.\;of\;pixels\;colocalizing\;in\;SHG\;and\;CARS\;channels}{Total\;number\;of\;pixels\;in\;SHG\;channel}$$ (2)

We also determined the number of SHG pixels not colocalizing with CARS. The term was normalized for each object using Eq. 3 by dividing with the total object pixel size. The total object pixel size refers to the total pixels, independent of the threshold of 25% of the maximum intensity.

$$Ratio\;ofSHG\;without\;CARS=\frac{Total\;number\;of\;pixels-pixels\;of\;CARS}{Total\;object\;pixel\;size}$$ (3)

## Results and discussion

For this proof-of-principle study, we chose two *S. cerevisiae* CEN. PK strains, BA6 and BA6∆, engineered to produce the lupane-type triterpenoids betulin, betulin aldehyde, and betulinic acid (Guo et al., 2022). In *S. cerevisiae*, the deletion of *PAH1*, encoding a phosphatidate phosphatase, was previously shown to increase the activity of phosphatidylserine synthase (PSS), which catalyzes the committed step for the synthesis of major phospholipids (Han and Carman, 2017). In strain BA6∆, the *PAH1* deletion was introduced to impair neutral lipid biosynthesis and enhance ER proliferation to foster triterpenoid accumulation (Adeyo et al., 2011; Walter, 2018). In both engineered strains constitutive downregulation of lanosterol synthase activity results in reduced ergosterol levels compared to the *S. cerevisiae* CEN. PK Guo et al., 2022. The altered lipophilic molecule profiles (lipids, sterols, triterpenoids) make these strains excellent test objects for evaluating techniques for analyzing the intracellular compositions in yeast.

### Nile red staining generated higher fluorescence signals in triterpenoid-producing strains than the reference

Nile red is a highly solvatochromic dye, which shows fluorescence in a hydrophobic environment (Tajalli et al., 2008) and been widely applied for microbial lipids determination (Brown et al., 1992; Morales-Palomo et al., 2022). Here, we aimed to compare fluorescence signals of engineered and reference yeast strains stained with Nile red to evaluate differences in the signal at different wavelengths. When dissolved in polar lipids such as phospholipids, the main components of lipid membranes, Nile red shows an intensely red fluorescence that shifts to bright yellow when dissolved in lipid droplets, mainly consisting of neutral lipids (triglycerides) (Greenspan et al., 1985). We used this stain to confirm differences in the hydrophobic compound composition of the chosen yeast strains, a prerequisite for CARS and SHG measurements. We measured the fluorescence at two different excitation and emission wavelengths: Ex/Em 515/583 nm for neutral lipids and 550/638 nm to detect polar phospholipids, as recommended by the manufacturer.

**FIGURE 3:**
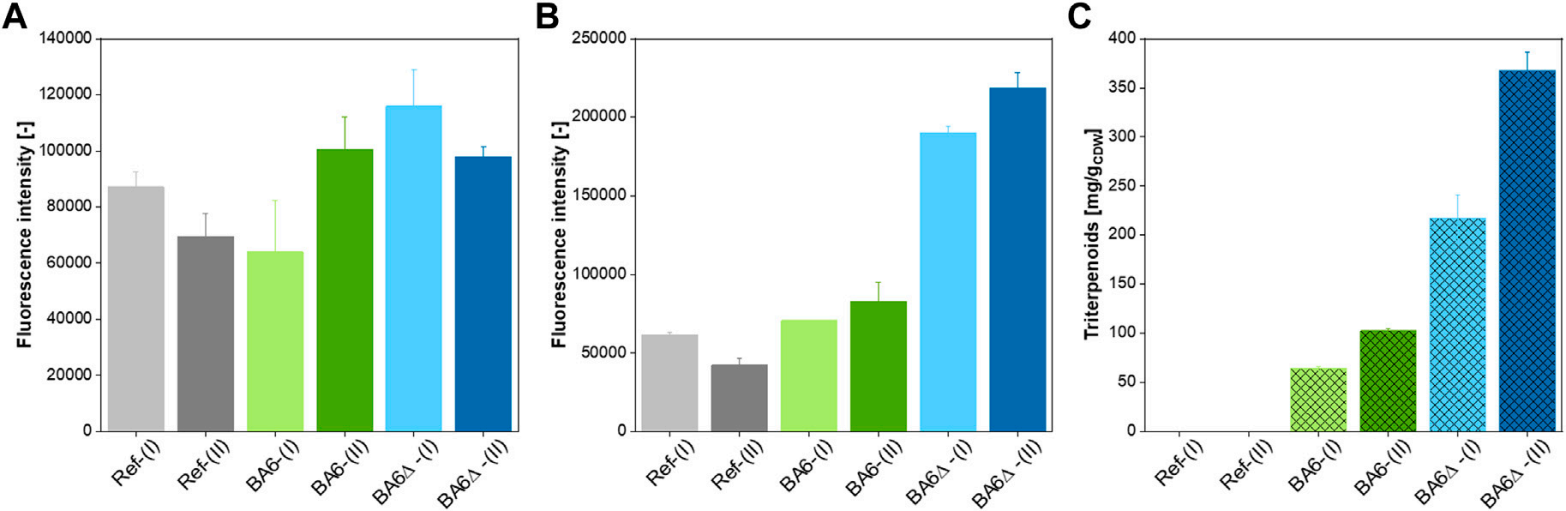
Qualitative determination of the hydrophobic molecule content of engineered yeast. **(A, B)**: Fluorescence intensity [-] at Ex/Em 515/583 nm **(A)**, 550/638 nm **(B)** of Nile red-stained cell suspensions of the reference strain (Ref), and the two betulinic acid-producing strains BA6 and BA6∆ sampled from a batch cultivation during growth on glucose (I) and after the diauxic shift during growth on ethanol (II). The OD_600_ of all samples was adjusted to 1. **(C)** Triterpenoid yield [mg/g_CDW_] of the reference strain (Ref) and the two betulinic acid-producing strains BA6 and BA6∆ sampled from a batch cultivation during growth on glucose (I) and after the diauxic shift during growth on ethanol (II).

The three yeast strains were cultivated under conditions optimized for triterpenoid production and harvested at two stages of the fermentation with low and high triterpenoid accumulation. If Nile red stains triterpenoids, cultures treated with this dye show distinctive fluorescence intensities. We indeed observed differences in fluorescence output in the microtiter plate assays of stained cell suspensions dependent on strain and fermentation stage. With measurements at 515/583 nm (Figure 3A), which mainly detects triglycerides and neutral lipids, no considerable difference between the reference and the engineered strains was observed, while at Ex/Em 550/638 nm (Figure 3B), the signal was considerably higher for the triterpenoid-producing strains. These differences can partially be related to the triterpenoid content of the cells, determined by HPLC (Figure 3C). Triterpenoids have a higher polarity than neutral lipids; hence might be detected at higher excitation wavelengths. Additionally, the higher fluorescence signal for the strain BA6∆ indicates higher phospholipid content, which is in line with the impaired neutral lipid biosynthesis and enhanced ER proliferation reported for *pah*1∆ mutants (Adeyo et al., 2011). Since this method falls short of distinguishing the different heterologous and native lipophilic compounds, more definitive explanations cannot be given.

### Triterpenoids and yeast-native ergosterol generate either SHG or CARS or both signals

Nile red staining indicated compositional differences between the reference and triterpenoid-producing yeast strains. Single-cell microscopy imaging techniques are required for subcellular localization of triterpenoids or time-resolved tracking of triterpenoid distribution in individual cells and molecule partitioning during cell proliferation. Here, we tested the applicability of CARS and SHG microscopy techniques as non-invasive methods for analyzing living yeast cells. First, we analyzed pure standards of triterpenoids and ergosterol to observe their detectability by CARS and SHG techniques. Ergosterol was included to estimate interference with triterpenoid signals because of its structural and biophysical similarity and the differential accumulation in the tested yeast strains.

**FIGURE 4:**
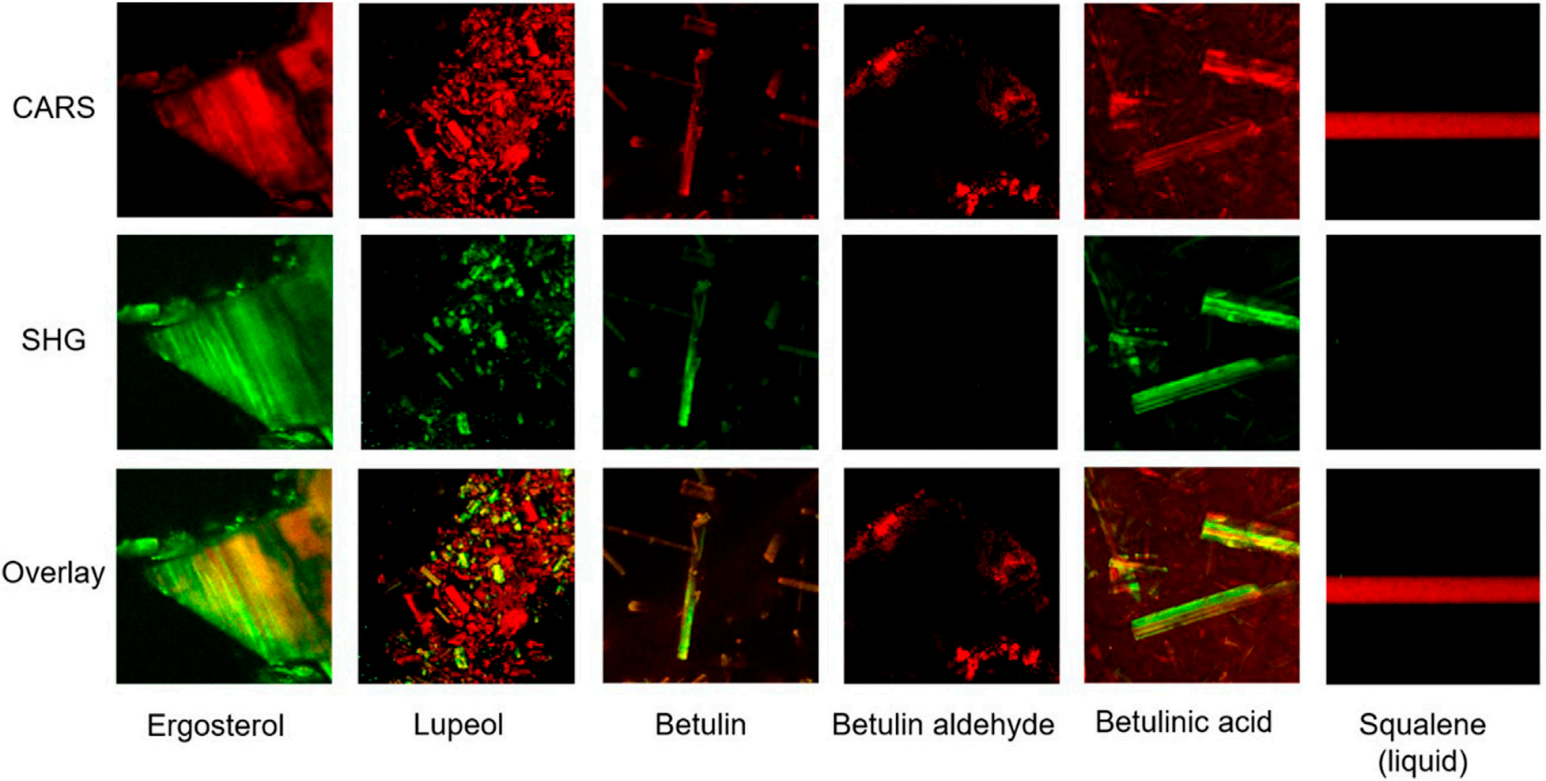
Evaluating CARS and SHG measurements for triterpenoid detection. CARS (red), SHG (green), and an overlay of both signals of solid ergosterol, lupeol, betulin, betulin aldehyde, and betulinic acid, and liquid squalene at 817 nm. Solid compounds were measured in xy and liquid squalene in xz dimension. Yellow areas in the overlay images emerge through colocalization of CARS and SHG signals, green or red are areas with solely or predominantly SHG or CARS signal, respectively; edge length = 75 µm.

SHG and CARS signals of crystalline ergosterol, lupeol, betulin, betulin aldehyde, betulinic acid, and liquid squalene were acquired at wavelengths between 807 nm and 819 nm (see Supplementary Figures S1, S2). While the SHG signal did not show any changes, the strongest CARS signal was observed at 816 nm and 817 nm; hence these wavelengths were chosen for further measurements. As described for structures with a high C-H content (Pezacki et al., 2011), all measured substances generated a CARS signal (Figure 4). In their crystalline state, ergosterol, lupeol, betulin, and betulinic acid were also SH-active, while no SHG signal was observed for betulin aldehyde. The overlay of both signals revealed a predominance of the SHG signal for ergosterol, betulin, and betulinic acid, while the opposite was seen for lupeol. As expected, the liquid squalene generated only a CARS but not an SHG signal, which is only generated by crystalline or ordered structures and not by fluids (Kleinman, 1962).

**FIGURE 5:**
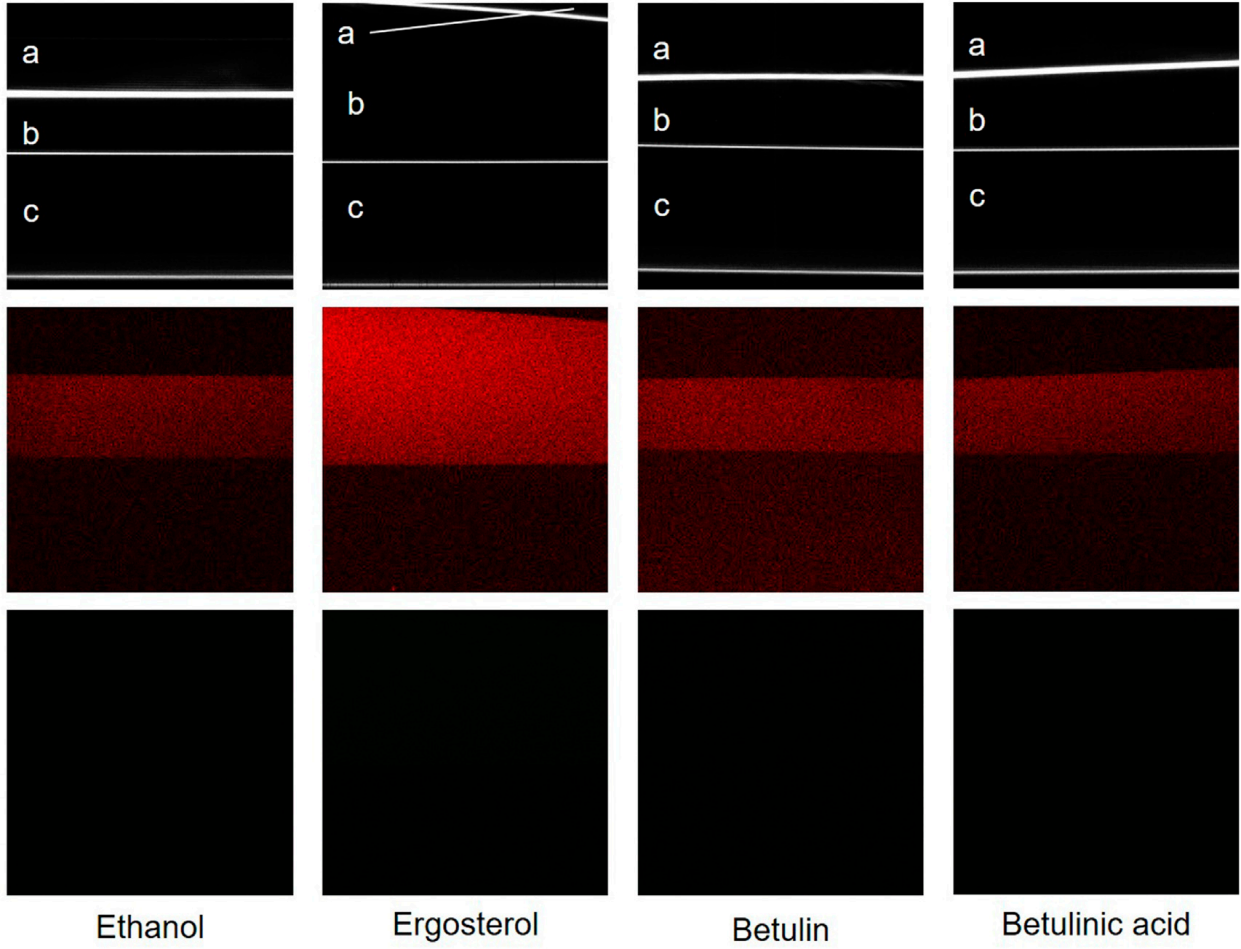
Transmission microscopy showing laser reflection (T) (white), CARS (red), and SHG (green) signals of pure ethanol and solutions of ergosterol, betulin, and betulinic acid in ethanol at 817 nm, measured in xz dimension; a: airside, b: sample, c: microscopy slide, edge length = 75 µm.

Likewise, ethanolic solutions of ergosterol, betulin, and betulinic acid showed a CARS signal but no SH activity (Figure 5).

### Spectroscopy allows non-invasive monitoring of intracellular triterpenoid accumulation

**FIGURE 6:**
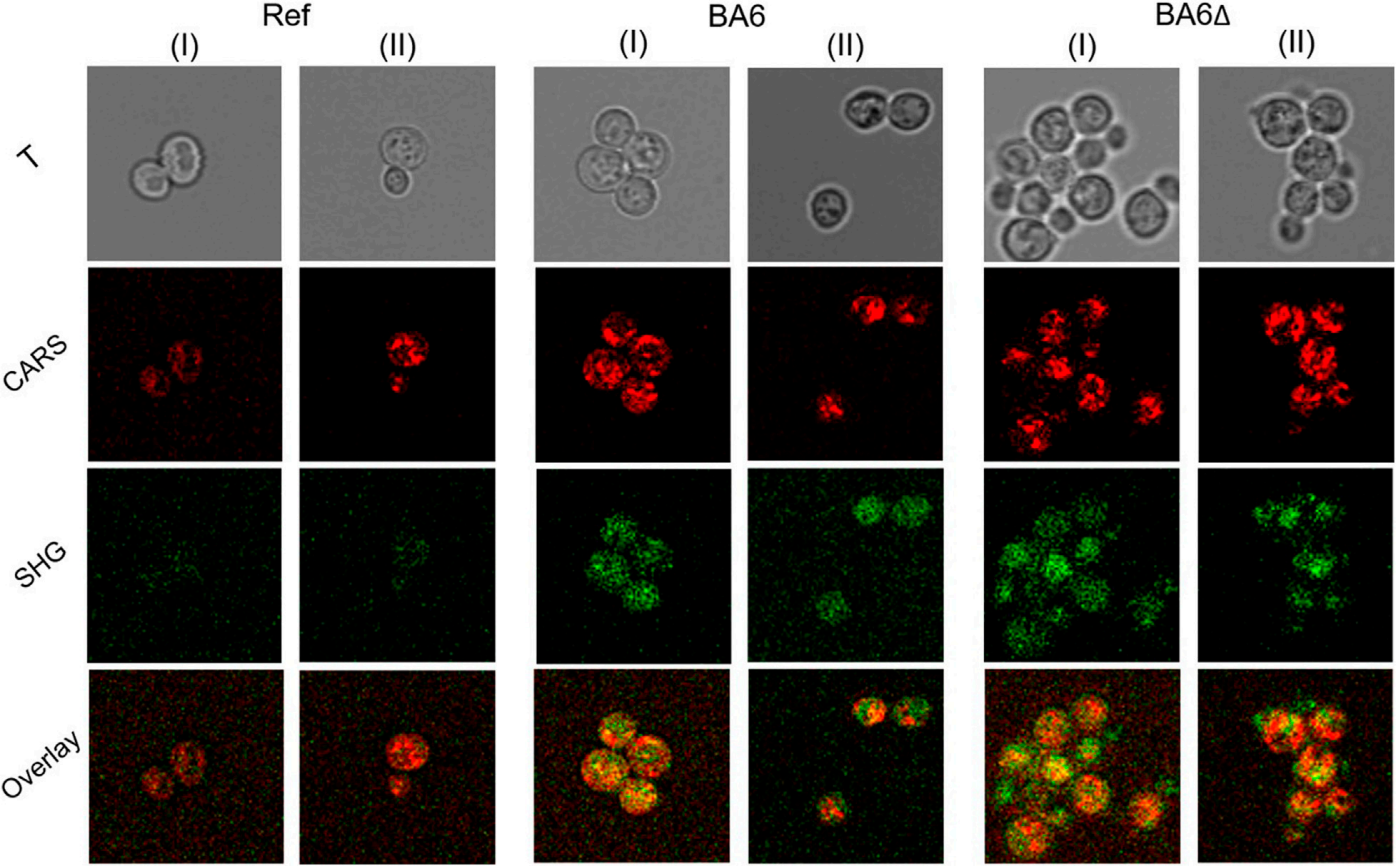
CARS and SHG measurements of triterpenoids in yeast. Transmission T (grey), CARS (red), SHG (green) signal, and the overlay of CARS and SHG signals for the reference (Ref) and triterpenoid producing strains BA6 and BA6∆ at early (I) and late fermentation stage (II). Measurements were performed at 816 nm; edge length is 25 µm.

To evaluate CARS and SHG microscopy for intracellular triterpenoid production in yeast cells, we compared batch cultures of the two triterpenoid-producing strains, BA6 and BA6∆, and the non-producing reference strain (Ref). Samples were taken at an early stage (I) and a late stage (II) of a batch cultivation with distinct changes in specific triterpenoid content (Table 1). Immobilized cells of the three strains were observed under the microscope; images were taken in transmission mode and CARS and SHG signals were recorded (Figure 6).

**TABLE 1:** Concentration of biomass, total triterpenoids (sum of betulinic acid, betulin, betulin aldehyde, and lupeol), and triterpenoid content of the investigated strains during growth in batch culture on glucose (I) and after the diauxic shift on ethanol (II).

Sample	CDW [g/L]	Total triterpenoid concentration [mg/L]
Ref-(I)	2.4 ± 0.0	—
Ref-(II)	6.9 ± 0.1	—
BA6-(I)	0.1 ± 0.0	98 ± 4
BA6-(II)	8.5 ± 0.5	1.061 ± 22
BA6Δ-(I)	0.2 ± 0.0	55 ± 1
BA6Δ-(II)	7.6 ± 0.4	1.520 ± 104

Both triterpenoid-producing strains showed a similar CARS signal at stage I, slightly stronger than the reference. C-H-containing compounds cause a strong CARS signal at 816 nm (El-Mashtoly et al., 2014) and represent native lipids as well as triterpenoids in the engineered strains. The triterpenoid-producing strains also generated substantial SHG signals. Although natively synthesized ergosterol (Figure 4) and the plasma membrane structure (Moen et al., 2014) can produce SHG, the SHG signal in the reference strain was noticeably lower than in the triterpenoid-producing strains. Together with the SHG signal of solid triterpenoid standards, the SH activity of BA6 and BA6∆ cells suggests that it is caused by triterpenoid agglomerates or (pseudo) crystals.

BA6∆ cells showed cell-to-cell heterogeneity in SHG, especially during stage I. Similarly, previous studies reported variability in endoplasmic reticulum morphology and lipid droplet abundance between single BA6∆ cells (Walter, 2018), which might therefore differ in lipid composition. However, if the here observed variability in SHG signal relates to this phenotype or differences in triterpenoid accumulation or lipid content cannot be stated.

Immobilized cells of the reference and engineered strains sampled during stage II showed comparable CARS signals. Spherical structures were detected by CARS in all three strains (Figure 6), which were presumed to be lipid droplets. Again, the SHG signal in triterpenoid-producing strains was much higher than in the non-producing strain.

The overlay of the CARS and SHG signals allows speculation of analyte distribution. While most regions show a colocalization (indicated by the yellow color), there are also regions with only or predominantly SHG or CARS activity, and this separation increased in stage II. Notably, areas of presumed lipid droplet localizations showed no SHG signal, suggesting that triterpenoids are either not present in these organelles or are not in a crystal-like form required for exerting SH activity. Contrary, regions with a predominant SH signal suggest the agglomeration of solid triterpenoid particles in a low-lipid environment.

**FIGURE 7:**
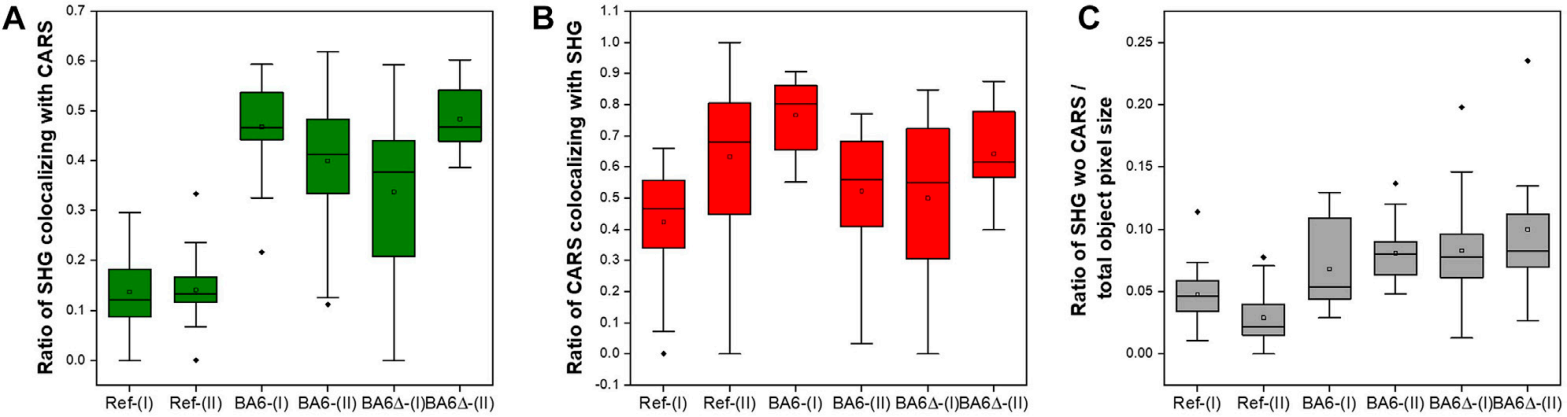
Box plot representation of colocalization analysis for the reference (Ref) and triterpenoid producing strains BA6 and BA6∆ during growth stage I and II. **(A)**: Ratio of SHG/CARS signal colocalization, **(B)**: Ratio of CARS signal colocalizing with SHG signal, **(C)**: Ratio of SHG signal not colocalizing with CARS normalized to the total object pixel size. Dots outside the box: Outliers; Vertical lines: Whiskers; Dots inside the box: Means; Horizontal line inside the box: median. The colocalization analysis was performed for single objects (representing single cells and in some cases cell agglomerates) in the microscopy image of each strain. The number of cells per image for the different strains was as follows: BA6 (I), 32 cells; BA6 (II), 24 cells; BA6Δ (I), 85 cells; BA6Δ (II), 58 cells; Ref (I), 58 cells; and Ref (II). 34 cells.

To further evaluate the localization of triterpenoids at the subcellular level, we performed a colocalization analysis, where we first determined the number of objects in each image, consisting of one or more cells. Then, the number of pixels above a threshold of 25% maximum intensity was determined for every object for CARS and SHG channels. Using these values, we determined ratios of colocalization as described in detail in the methods section. The ratio of pixels with CARS signal, which colocalize with SHG (Figure 7A), was in a similar range for all strains in both stages. Importantly, it showed that CARS signal does not occur in all areas where SHG signal was detected as expected, emphasizing the validity of this data evaluation approach. The SHG signal colocalizing with CARS showed a trend towards higher values in triterpenoid-producing strains than in the reference (Figure 7B). However, the microscopy data also showed a higher fraction of pixels with SHG signal for the engineered strains (∼20–30%) compared to the reference strain (<10%) and that the fraction of pixels with SHG signal non-colocalized with CARS over all pixels per object (Figure 7C) was increased in the engineered strains. The overlay of CARS and SHG of solid sterols/terpenoids shows a predominance of SHG at least for some compounds. Hence pixels with SHG non-colocalized with CARS point to the enrichment of these molecules in solid/pseudocrystal form and in a low-lipid environment. Assuming that triterpenoids, when located in lipid membranes and lipid droplets, always result in both CARS and SHG signals, our results hypothesize that triterpenoids might also accumulate in other cellular spaces or be enriched in microdomains (lipid raft-like structures) within membranes.

The large variance in some data sets indicated by the whiskers and outliers may have been caused by biological heterogeneity between individual cells and analytical challenges when cell agglomerates are formed. Despite the significant variance, and important for the scope of this study, these results indicate a correlation of SHG signal with triterpenoid accumulation and the subcellular distribution.

Overall and important for the scope of this study, these results indicate a correlation of SHG signal with triterpenoid accumulation and the subcellular distribution.

### Online monitoring indicates population heterogeneity of triterpenoid production

**FIGURE 8:**
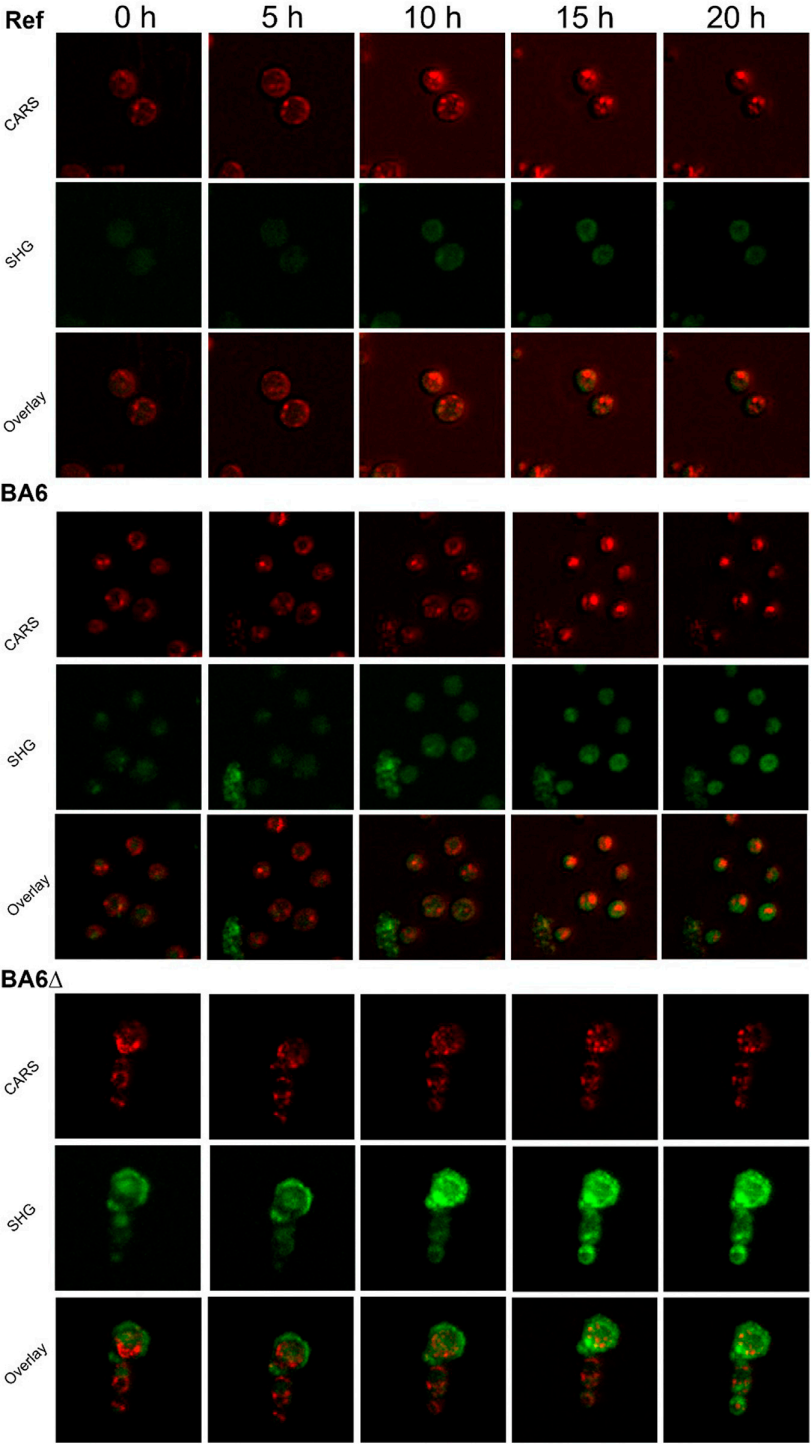
Time resolved triterpenoid production in yeast. Measurement of CARS (red) and SHG (green) signals and the overlay for the reference strain (Ref), and triterpenoid producing strains BA6 and BA6∆ during growth on ethanol in a static-culture. Overlay images show yellow in areas of strong colocalization of CARS and SHG. Cultivation took place at room temperature. The culture was aerated with a water and ethanol solution (50:50 v/v). Measurements were performed at 816 nm; edge length = 25 µm.

Online measurements of the reference strain and triterpenoid-producing yeast strains (Figure 8) allowed us to monitor dynamic changes in CARS and SHG signals during yeast cultivation. Note that the real-time data (Figure 8) represent the maximum intensity projection of the signal measured for nine levels in *z*-direction. This way, the sensitivity was increased and importantly, consistent values were achieved even with vertical movements of the living cells. This improved analytical method allowed better capture of the SHG signal of the reference cells, which increased over time. The cultivations were performed with ethanol as sole carbon source at initial concentrations of 16 g/L. It is known that elevated ethanol concentrations induce ergosterol production in yeast (Swan and Watson, 1998). The potentially higher ergosterol content could also have contributed to the higher SHG signal excreted by cells under these growth conditions compared to cells from glucose batch cultivations.

Also, considerable differences in the CARS and SHG signals were observed throughout the cultivation of the triterpenoid-producing strains BA6 and BA6∆. The SHG signal increased in both cultures over time, indicating accumulation of triterpenoids. While the SHG signal in BA6 was nearly equal in all cells, for BA6∆, some differences were observed in the SHG signal of individual cells. Figure 8 shows 3 cells of BA6∆ that form a cluster. They differ in size and SHG signal. The larger size of the upper cell suggests it to be an old cell, explaining its high SHG signal from the beginning. Contrary, the two smaller cells had a weak SHG signal at the beginning, which increased over time, likely correlating with intracellular triterpenoid accumulation. The overlay of SHG and CARS pictures revealed no or very few colocalizations. Again, indicating that the triterpenoids were mostly not present in soluble form in a lipid rich environment. Whether they are locally enriched in lipid membranes or even present in an aqueous surrounding, e.g., the vacuole cannot be distinguished with the current measurements.

One limitation of the here presented methodology is that CARS also detects triterpenoids (Yu et al., 2008), hence, the signals of both methods should be considered for a deeper evaluation of the results. These data show clear differences in producing and non-producing strains and importantly allow time-resolved observation on single cell level—the greatest advantages of the applied non-invasive microscopy technique compared to analytical methods, such as HPLC, which provide an averaged triterpenoid concentration of an entire population.

## Conclusion

In this proof of principle study, we used three techniques (Nile red staining, CARS and SHG microscopy) to analyze lipid compounds and triterpenoids in yeast cells. We could show that the combination of the non-invasive techniques CARS and SHG allow detection of lipids and triterpenoids in living yeast cells. This highlights the great potential of these methods for future analysis of natural product synthesis with special and temporal resolution. Other than conventional methods like Nile red, the application of CARS and SHG allows the detection of both triterpenoids and lipids in individual cells. The application of CARS and SHG signal measurement shows potential for further development and an advanced quantification. For instance, detection of the individual triterpenoid components at different wavelengths would be an option to further improve the existing method towards more specific and quantitative analysis. Using the current state-of-the art techniques, we were able to monitor triterpenoid accumulation in cells of a population over the entire cultivation time. To our knowledge, this is the first time that the combination of both CARS and SHG techniques are applied for the analysis of intracellular product accumulation in living yeast cells. This proof-of-principle study shows the potential of application of CARS and SHG microscopy techniques in applied microbiology allowing new insights into the dynamics of triterpenoid production and storage by recombinant yeasts.

## Data Availability

The raw data supporting the conclusion of this article will be made available by the authors, without undue reservation.
