# Supplementary material for: Non-invasive monitoring of microbial triterpenoid production using nonlinear microscopy techniques
Source: Front Bioeng Biotechnol. 2023 Feb 28;11:1106566. doi: 10.3389/fbioe.2023.1106566 (PMC10012247; doi:10.3389/fbioe.2023.1106566)
Supplement: Supplementary file 1 [file DataSheet1.DOCX]

Supplementary Material
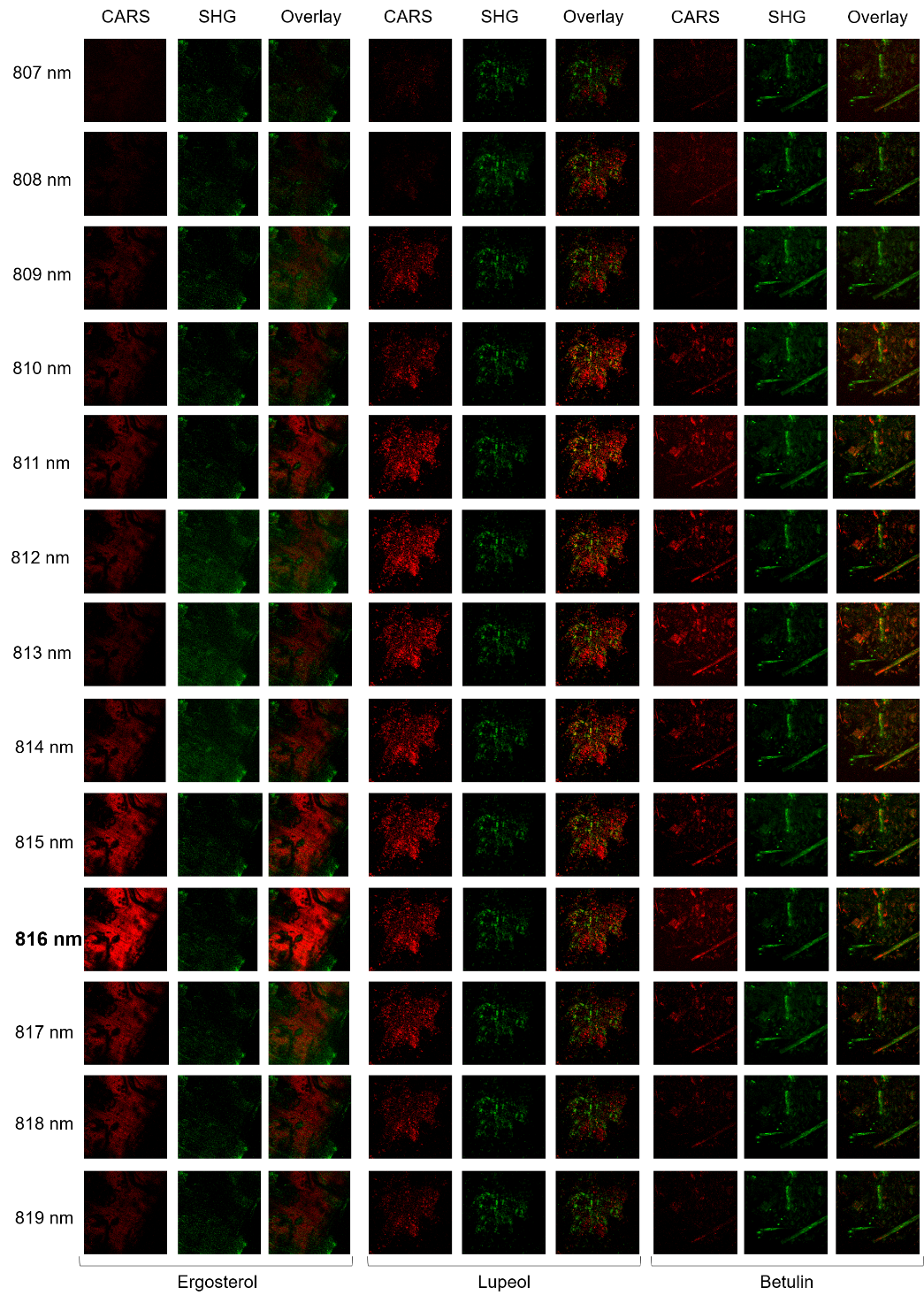


Supplementary Figure 1: CARS (red), SHG (green), and an overlay of both signals of solid ergosterol, lupeol, and botulin at different wavelengths between 807 nm and 819 nm. Solid compounds were measured in xy dimension. Yellow areas in the overlay images emerge through colocalization of CARS and SHG signals; green or red are areas with solely or predominantly SHG or CARS signals, respectively; edge length = 100 µm.


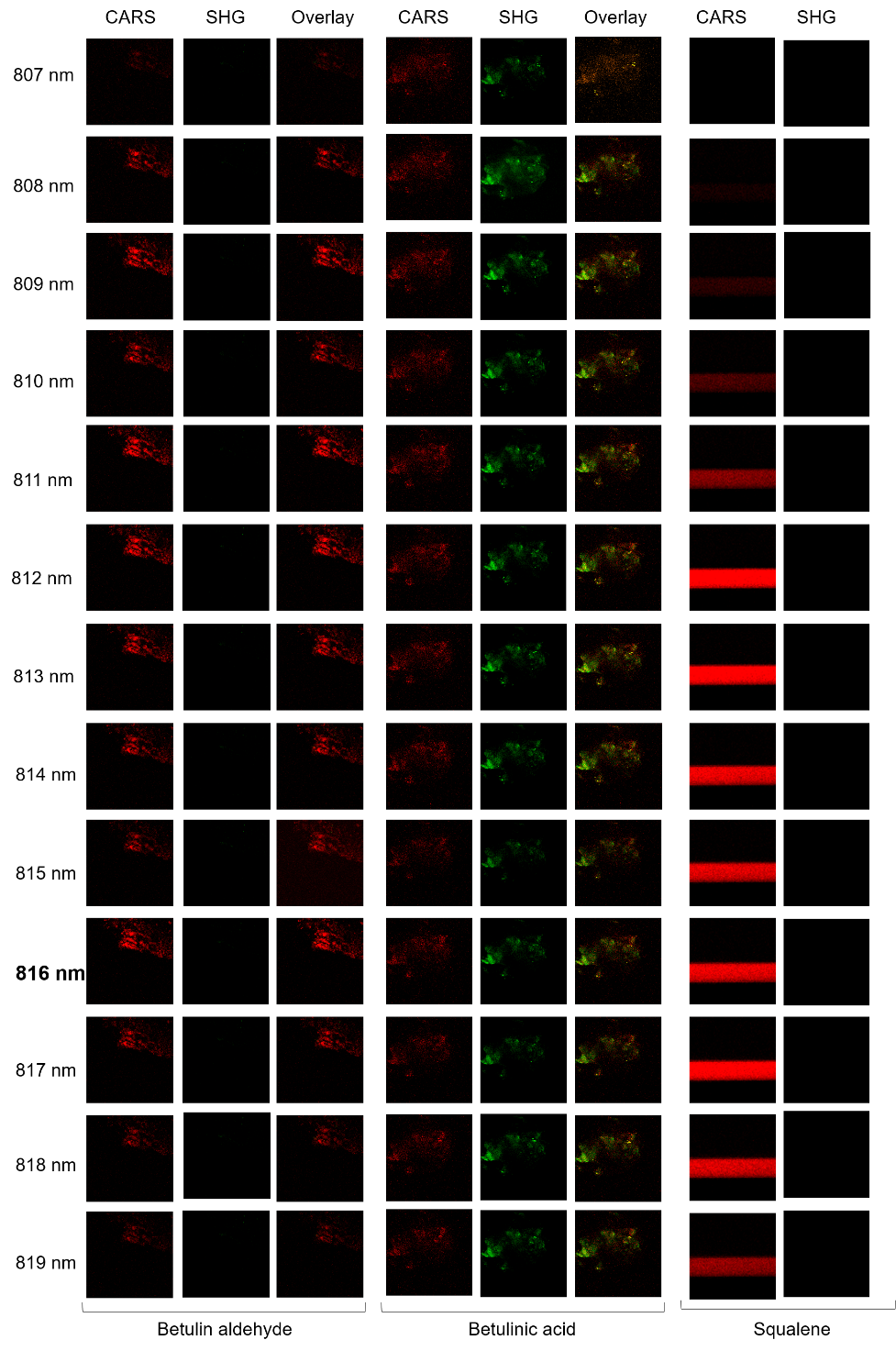


Supplementary Figure 2: CARS (red), SHG (green), and an overlay of both signals of solid betulin aldehyde and betulinic acid, and CARS (red), SHG (green) of liquid squalene at different wavelengths between 807 nm and 819 nm. Solid compounds were measured in xy and liquid squalene in xz dimension. Yellow areas in the overlay images emerge through colocalization of CARS and SHG signals; green or red are areas with solely or predominantly SHG or CARS signal, respectively; images of betulin aldehyde and betulinic acid edge length = 100 µm, images of squalene; edge length = 50 µm.
